# *cyclo*-Tetra-μ-fluorido-1:2κ^2^ *F*;2:3κ^2^ *F*;3:4κ^2^ *F*;1:4κ^2^ *F*-octa­nitrato-1κ^8^ *O*,*O*′;3κ^8^ *O*,*O*′-tetra­kis­(1,10-phenanthroline)-2κ^4^ *N*,*N*′;4κ^4^ *N*,*N*′-2,4-dichromium(III)-1,3-dineodymium(III) methanol tetra­solvate monohydrate

**DOI:** 10.1107/S1600536811042383

**Published:** 2011-10-22

**Authors:** Torben Birk, Magnus Schau-Magnussen, Thomas Weyhermüller, Jesper Bendix

**Affiliations:** a Department of Chemistry, University of Copenhagen, Universitetsparken 5, DK-2100 Copenhagen, Denmark; b MPI für Bioanorganische Chemie, Stiftstrasse 34–36, PO Box 101365, D-45413 Mülheim an der Ruhr, Germany

## Abstract

In the title compound, [Cr_2_Nd_2_F_4_(NO_2_)_8_(C_12_H_8_N_2_)_4_]·4CH_3_OH·H_2_O, two *cis*-difluoridobis(1,10-phenanthroline)chromium(III) fragments containing octa­hedrally coordinated chromium(III) bridge *via* fluoride ions to two tetra­nitratoneodymate(III) fragments, forming an uncharged tetra­nuclear square-like core. The fluoride bridges are fairly linear, with Cr—F—Nd angles of 168.74 (8)°. Cr—F bond lengths are 1.8815 (15) Å, slightly elongated compared to those of the parent chromium(III) complex, which has bond lengths ranging from 1.8444 (10) to 1.8621 (10) Å. The tetra­nuclear complex is centered at a fourfold rotoinversion axis, with the Cr and Nd atoms situated on two perpendicular twofold rotation axes. The uncoordinated water mol­ecule resides on a fourfold rotation axis. The four methanol solvent mol­ecules are located around this axis, forming a cyclic hydrogen-bonded arrangement. The title compound is the first structurally characterized example of unsupported fluoride bridges between lanthanide and transition metal ions.

## Related literature

For related structures of second sphere inter­actions with robust chromium(III) fluoride complexes, see: Birk *et al.* (2010 ▶); Terasaki *et al.* (1999 ▶); Kaizaki & Takemoto (1990 ▶). For other examples of fluoride bridges between 3*d* and 4*f* metal atoms, see: Pevec *et al.* (2003 ▶); McRobbie *et al.* (2011 ▶). For the structure of the cationic chromium precursor complex, see: Birk *et al.* (2008 ▶). For the synthesis of the precursor, see: Glerup *et al.* (1970 ▶). For importance of the title compound in the context of magnetic materials, see: Kahn (1985 ▶, 1987 ▶); Sessoli & Powell (2009 ▶). For crystallographic background, see: Coppens (1970 ▶).
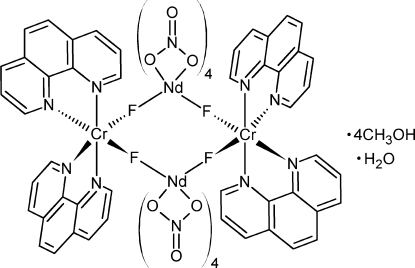


## Experimental

### 

#### Crystal data

[Cr_2_Nd_2_F_4_(NO_2_)_8_(C_12_H_8_N_2_)_4_]·4CH_4_O·H_2_O
*M* *_r_* = 1831.56
Tetragonal, 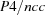

*a* = 17.632 (4) Å
*c* = 20.955 (3) Å
*V* = 6515 (2) Å^3^
*Z* = 4
Mo *K*α radiation
μ = 2.01 mm^−1^
*T* = 122 K
0.35 × 0.29 × 0.24 mm

#### Data collection

Nonius KappaCCD area-detector diffractometer
Absorption correction: integration (Gaussian; Coppens, 1970 ▶) *T*_min_ = 0.601, *T*_max_ = 0.718
339826 measured reflections
10126 independent reflections
6979 reflections with *I* > 2σ(*I*)
*R*_int_ = 0.047

#### Refinement

*R*[*F* ^2^ > 2σ(*F* ^2^)] = 0.036
*wR*(*F* ^2^) = 0.102
*S* = 1.27
10126 reflections
239 parameters
H-atom parameters constrained
Δρ_max_ = 2.41 e Å^−3^
Δρ_min_ = −1.78 e Å^−3^

### 

Data collection: *COLLECT* (Nonius, 1999 ▶); cell refinement: *COLLECT*; data reduction: *EVALCCD* (Duisenberg *et al.*, 2003 ▶); program(s) used to solve structure: *SHELXS97* (Sheldrick, 2008 ▶); program(s) used to refine structure: *SHELXL97* (Sheldrick, 2008 ▶); molecular graphics: *ORTEP-3* (Farrugia, 1997 ▶) and *Mercury* (Macrae *et al.*, 2006 ▶); software used to prepare material for publication: *SHELXL97*.

## Supplementary Material

Crystal structure: contains datablock(s) global, I. DOI: 10.1107/S1600536811042383/wm2538sup1.cif

Structure factors: contains datablock(s) I. DOI: 10.1107/S1600536811042383/wm2538Isup2.hkl

Additional supplementary materials:  crystallographic information; 3D view; checkCIF report

## Figures and Tables

**Table 1:** Hydrogen-bond geometry (Å, °)

*D*—H⋯*A*	*D*—H	H⋯*A*	*D*⋯*A*	*D*—H⋯*A*
O20—H20⋯O20^i^	0.84	1.89	2.700 (4)	161

Symmetry code: (i) 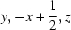.

**Fig. 1.**
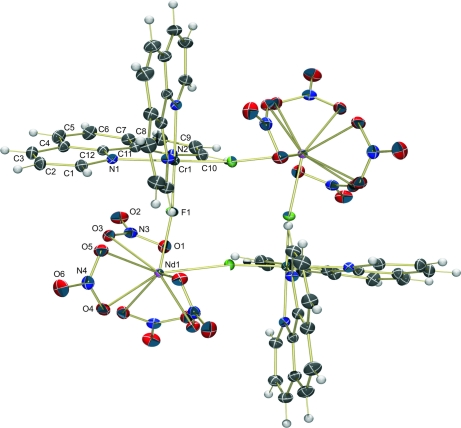
A view of the tetranuclear molecular structure of the title compound with the atom-labelling scheme. Displacement ellipsoids are drawn at the 50% probability level. Solvent methanol and water molecules were omitted.

**Fig. 2.**
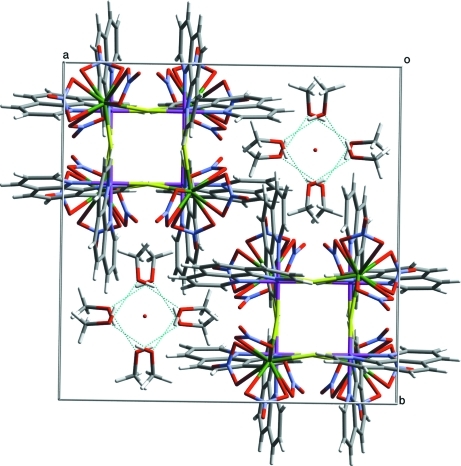
A packing diagram in projection along [001] showing hydrogen bonds between the methanol solvent molecules (dotted lines).

## supplementary crystallographic information

### Comment

The magnetic properties of polynuclear, mixed lanthanoide transition metal complexes have received much attention (Sessoli & Powell, 2009). Since early suggestions by Kahn (1985, 1987) that exchange interactions involving *d*- and *f*-electrons were likely to lead to ferromagnetic coupling, due to vanishing orbital overlaps, many such systems have been synthesized and studied structurally and magnetically. Despite the high activity in this field, there are still simple types of bridging ligands, which have not been studied in this context. Thus, fluoride, which is known to bind strongly to lanthanoides has not been known as a bridging ligand between paramagnetic transition metal ions and lanthanoide ions until the very recent introduction of fluoride in heterometallic wheels by McRobbie *et al.* (2011). However, in those systems, fluoride bridges are always supported by carboxylate groups connecting the same metal ions. Based on those systems it is very difficult or impossible to make deductions concerning the geometric preferences of fluoride as a bridging ion and concerning magnetic exchange over fluoride bridges. This problem is remedied by a system such as the title compound, which is the first example of unsupported fluoride bridges between 3*d* and 4*f* metals.

In the title compound the solvate water molecule is located on a proper fourfold axis, whereas the tetranuclear Cr_2_Nd_2_F_4_ fragment is centered on a fourfold rotoinversion axes. Consequently, all the metal ions are required to lie in the same plane perpendicular to the tetragonal axes (Fig. 1). The complexation of the neodymium atom induces a slight elongation of the Cr—F bonds by *ca* 0.03 Å in comparison with the parent compound (Birk *et al.*, 2008). The neodymium atom is 10-coordinated with its coordination sphere completed by bidentate nitrate ions coordinating with unexceptional bond lengths and bite angles. The uncoordinated water molecule is located on a fourfold axis and has no direct partner for hydrogen bonding (the next nearest atom is C5 in a distance of 3.816 (3) Å), which explains the high thermal displacement parameters for its oxygen atom. Around the same fourfold axis, the methanol solvate molecules form a cyclic tetrameric arrangement held together by hydrogen bonds (Table 1, Fig. 2).

Studies of the magnetic properties of this system and the possible generalization of this route to fluoride-bridged systems are currently being undertaken.

Related structures of second sphere interactions with robust chromium(III) fluoride complexes were presented by Birk *et al.* (2010); Terasaki *et al.* (1999); Kaizaki & Takemoto (1990). For other examples of fluoride bridges between 3*d* and 4*f* metal atoms, see: Pevec *et al.* (2003).

### Experimental

*trans*-[Cr(py)_4_F_2_]NO_3_ is synthesized by the literature method (Glerup *et al.*, 1970). 1,10-phenantroline (Alfa Aesar), Nd(NO_3_)_3_^.^6H_2_O (Alfa Aesar; 99.9%), 2-methoxyethanol (Sigma-Aldrich; 99.3+%) and methanol (Lab-Scan; Anhydroscan) were all used as received. The synthesis of *cis*-[Cr(phen)_2_F_2_]NO_3_ proceeds in many ways analogous to the method described by Glerup *et al.* (1970) for the synthesis of *cis*-[Cr(phen)_2_F_2_]ClO_4_. As a result of a significant difference in solubility of the two salts, some modification with respect to solvent volume and isolation procedure has been introduced. It should also be noted that the nitrate can be crystallized with a variable number of crystal water and that this number can change depending on whether the substance is stored in dry or moist air. Elemental analysis for C, H and N was performed with an CE Instrument: FLASH 1112 series EA, at the microanalytic laboratory, University of Copenhagen. Electrospray (ES) mass spectra were recorded on a Micromass Q-TOF apparatus with positive ion detection.

i) Syntesis of the starting material *cis*-[Cr(phen)_2_F_2_]NO_3_

*trans*-[Cr(py)_4_F_2_]NO_3_ + 1,10-phenantroline = *cis*-[Cr(phen)_2_F_2_]NO_3_ + 4py

*trans*-[Cr(py)_4_F_2_]NO_3_ (36.7 g; 0.078 mol) and 1,10-phenantroline (34.8 g; 0.19 mol) were placed in a conical flask (500 ml) with 2-methxyethanol (250 ml). The mixture was heated to boiling temperature, whereby a violet solution formed, followed shortly by precipitation of a red-violet solid. The heating was continued for 1 h followed by cooling to room temperature before a purple red product was isolated. The raw product was washed with ethanol (2x100 ml) and dried by suction.

Yield of raw product: 31.7 g (79.0% of theoretical based on Cr^III^). Analysis: Calcd. for H_17_C_24_N_5_O_4_F_2_Cr_1_: H, 3.29%; C, 55.28%; N, 13.43%. Found: H, 3.16%; C, 55.25%; N, 13.30% (sesqui hydrat). TOF MS ES^+^ (MeOH): m/*z*: 450.5 ([Cr(phen)_2_F_2_]^+^)

ii) Syntesis of the title compound {[Cr(phen)_2_(µ-F)_2_][Nd(NO_3_)_4_]}_2_^.^CH_3_OH^.^H_2_O

2 *cis*-[Cr(phen)_2_F_2_]NO_3_ + 2 Nd(NO_3_)_3_ = {[Cr(phen)_2_(µ-F)_2_][Nd(NO_3_)_4_]}_2_

The title compound was prepared by reaction of a methanolic solution of *cis*-[Cr(phen)_2_F_2_](NO_3_) (210 mg, 0.41 mmol in 10 ml) with a methanolic solution of Nd(NO_3_)_3_^.^6H_2_O (175 mg, 0.40 mmol in 5 ml). Before combination, both solutions were filtered through filters with pore size 0.45 µm. Crystals formed over a period of 2–12 h. The yield was 284 mg (82% based on Nd). Crystals suitable for single-crystal X-ray diffraction were obtained directly using the concentrations given above. Upon drying, the crystals loose solvent and deteriorate. For the diffraction experiment, a crystal was taken from the mother liquor, covered with paraffin oil and cooled directly.

### Refinement

H atoms were found in a difference Fourier map and were included in the refinement as constrained idealized protons riding the parent atom, with *X*—H = 0.84 Å (OH); 0.95 Å (aromatic CH); 0.98 Å (CH_3_) with *U*_iso_ equal to 1.2×*U*_eq_ of the parent C atom (1.5×*U*_eq_ of the parent atom in MeOH). No resonable assignment of the H atoms of the water of crystallization could be obtained. Consequently, these H atoms were excluded from the refinement. The maximum residual electron density is found at 1.04 Å from O20, the minimum residual electron density is at 0.37 Å from the same atom.

### Crystal data

**Table:**

[Cr_2_Nd_2_F_4_(NO_2_)_8_(C_12_H_8_N_2_)_4_]·4CH_4_O·H_2_O	*D*_x_ = 1.867 Mg m^−^^3^
*M**_r_* = 1831.56	Mo *K*α radiation, λ = 0.71073 Å
Tetragonal, *P*4/*n**c**c*	Cell parameters from 120466 reflections
Hall symbol: -P 4a 2ac	θ = 2.3–40.1°
*a* = 17.632 (4) Å	µ = 2.01 mm^−^^1^
*c* = 20.955 (3) Å	*T* = 122 K
*V* = 6515 (2) Å^3^	Prism, pink
*Z* = 4	0.35 × 0.29 × 0.24 mm
*F*(000) = 3640	

### Data collection

**Table:**

Nonius KappaCCD area-detector diffractometer	10126 independent reflections
Radiation source: fine-focus sealed tube	6979 reflections with *I* > 2σ(*I*)
graphite	*R*_int_ = 0.047
ω and φ scans	θ_max_ = 40.1°, θ_min_ = 2.3°
Absorption correction: integration (Gaussian; Coppens, 1970)	*h* = −31→31
*T*_min_ = 0.601, *T*_max_ = 0.718	*k* = −29→31
339826 measured reflections	*l* = −37→37

### Refinement

**Table:**

Refinement on *F*^2^	Primary atom site location: structure-invariant direct methods
Least-squares matrix: full	Secondary atom site location: difference Fourier map
*R*[*F*^2^ > 2σ(*F*^2^)] = 0.036	Hydrogen site location: inferred from neighbouring sites
*wR*(*F*^2^) = 0.102	H-atom parameters constrained
*S* = 1.27	*w* = 1/[σ^2^(*F*_o_^2^) + (0.0144*P*)^2^ + 22.4316*P*] where *P* = (*F*_o_^2^ + 2*F*_c_^2^)/3
10126 reflections	(Δ/σ)_max_ = 0.002
239 parameters	Δρ_max_ = 2.41 e Å^−^^3^
0 restraints	Δρ_min_ = −1.78 e Å^−^^3^

### Special details

**Table:**

Geometry. All esds (except the esd in the dihedral angle between two l.s. planes) are estimated using the full covariance matrix. The cell esds are taken into account individually in the estimation of esds in distances, angles and torsion angles; correlations between esds in cell parameters are only used when they are defined by crystal symmetry. An approximate (isotropic) treatment of cell esds is used for estimating esds involving l.s. planes.
Refinement. Refinement of *F*^2^ against ALL reflections. The weighted *R*-factor *wR* and goodness of fit *S* are based on *F*^2^, conventional *R*-factors *R* are based on *F*, with *F* set to zero for negative *F*^2^. The threshold expression of *F*^2^ > 2*σ*(*F*^2^) is used only for calculating *R*-factors(gt) *etc*. and is not relevant to the choice of reflections for refinement. *R*-factors based on *F*^2^ are statistically about twice as large as those based on *F*, and *R*-factors based on ALL data will be even larger.

### Fractional atomic coordinates and isotropic or equivalent isotropic displacement parameters (Å^2^)

**Table:**

	*x*	*y*	*z*	*U*_iso_*/*U*_eq_	
Nd1	0.120550 (5)	0.879450 (5)	0.2500	0.01191 (3)	
Cr1	0.142468 (17)	0.642468 (17)	0.2500	0.01199 (6)	
F1	0.14142 (8)	0.74891 (8)	0.24362 (7)	0.0179 (2)	
N1	0.14262 (10)	0.52607 (10)	0.24070 (8)	0.0148 (3)	
N2	0.13452 (11)	0.63381 (11)	0.15243 (8)	0.0154 (3)	
N3	0.05724 (12)	0.80814 (11)	0.36783 (10)	0.0194 (3)	
N4	−0.00247 (12)	0.89135 (13)	0.15390 (10)	0.0200 (3)	
O1	0.12382 (11)	0.83605 (11)	0.36641 (9)	0.0224 (3)	
O2	0.01948 (10)	0.81071 (10)	0.31637 (8)	0.0194 (3)	
O3	0.03125 (13)	0.77993 (12)	0.41658 (9)	0.0280 (4)	
O4	0.02408 (11)	0.94903 (11)	0.18224 (10)	0.0237 (3)	
O5	0.01769 (11)	0.82711 (11)	0.17550 (9)	0.0227 (3)	
O6	−0.04596 (12)	0.89763 (14)	0.10853 (9)	0.0297 (4)	
C1	0.15642 (14)	0.47330 (13)	0.28428 (11)	0.0186 (3)	
H1	0.1678	0.4886	0.3267	0.022*	
C2	0.15473 (15)	0.39539 (14)	0.26990 (12)	0.0222 (4)	
H2	0.1663	0.3590	0.3019	0.027*	
C3	0.13624 (15)	0.37223 (13)	0.20931 (12)	0.0217 (4)	
H3	0.1327	0.3197	0.1996	0.026*	
C4	0.12255 (14)	0.42689 (13)	0.16182 (11)	0.0193 (4)	
C5	0.10393 (16)	0.40922 (15)	0.09673 (12)	0.0248 (4)	
H5	0.0975	0.3577	0.0845	0.030*	
C6	0.09540 (17)	0.46455 (16)	0.05244 (12)	0.0258 (5)	
H6	0.0818	0.4514	0.0100	0.031*	
C7	0.10658 (14)	0.54258 (14)	0.06875 (11)	0.0203 (4)	
C8	0.10612 (16)	0.60257 (16)	0.02423 (11)	0.0235 (4)	
H8	0.0953	0.5929	−0.0194	0.028*	
C9	0.12147 (16)	0.67492 (16)	0.04442 (12)	0.0250 (4)	
H9	0.1227	0.7154	0.0145	0.030*	
C10	0.13538 (15)	0.68919 (14)	0.10924 (11)	0.0206 (4)	
H10	0.1457	0.7396	0.1226	0.025*	
C11	0.12150 (12)	0.56114 (12)	0.13254 (10)	0.0159 (3)	
C12	0.12809 (12)	0.50311 (12)	0.17959 (10)	0.0150 (3)	
O20	0.14380 (18)	0.22887 (17)	0.41608 (17)	0.0543 (8)	
H20	0.1608	0.2733	0.4185	0.081*	
C20	0.07522 (19)	0.22365 (18)	0.45093 (15)	0.0315 (6)	
H20A	0.0838	0.2404	0.4949	0.047*	
H20B	0.0576	0.1709	0.4510	0.047*	
H20C	0.0367	0.2560	0.4310	0.047*	
O30	0.2500	0.2500	0.0863 (5)	0.225 (9)	

### Atomic displacement parameters (Å^2^)

**Table:**

	*U*^11^	*U*^22^	*U*^33^	*U*^12^	*U*^13^	*U*^23^
Nd1	0.01150 (4)	0.01150 (4)	0.01274 (5)	0.00073 (4)	−0.00057 (3)	−0.00057 (3)
Cr1	0.01221 (9)	0.01221 (9)	0.01155 (14)	−0.00036 (12)	0.00017 (10)	−0.00017 (10)
F1	0.0179 (5)	0.0122 (5)	0.0237 (6)	0.0010 (4)	0.0006 (5)	−0.0002 (5)
N1	0.0151 (7)	0.0146 (6)	0.0147 (7)	−0.0010 (5)	0.0001 (5)	0.0003 (5)
N2	0.0189 (8)	0.0152 (7)	0.0120 (5)	−0.0002 (5)	0.0008 (6)	0.0010 (5)
N3	0.0232 (9)	0.0175 (8)	0.0176 (7)	0.0015 (6)	0.0030 (6)	−0.0008 (6)
N4	0.0179 (8)	0.0260 (9)	0.0160 (7)	0.0005 (7)	−0.0011 (6)	−0.0001 (6)
O1	0.0222 (8)	0.0268 (8)	0.0181 (7)	−0.0014 (6)	−0.0026 (6)	0.0010 (6)
O2	0.0188 (7)	0.0216 (7)	0.0178 (7)	−0.0028 (6)	0.0004 (5)	0.0008 (6)
O3	0.0395 (11)	0.0260 (9)	0.0183 (7)	−0.0027 (8)	0.0073 (7)	0.0043 (6)
O4	0.0230 (8)	0.0207 (7)	0.0274 (8)	0.0029 (6)	−0.0073 (7)	−0.0023 (6)
O5	0.0242 (8)	0.0210 (8)	0.0229 (8)	−0.0026 (6)	−0.0054 (6)	0.0011 (6)
O6	0.0282 (9)	0.0411 (12)	0.0198 (8)	0.0035 (8)	−0.0104 (7)	0.0011 (8)
C1	0.0219 (9)	0.0178 (8)	0.0161 (8)	0.0003 (7)	0.0010 (7)	0.0025 (7)
C2	0.0267 (11)	0.0171 (9)	0.0229 (9)	0.0001 (8)	0.0008 (8)	0.0056 (8)
C3	0.0255 (10)	0.0157 (8)	0.0238 (9)	−0.0012 (7)	0.0013 (8)	0.0002 (7)
C4	0.0208 (9)	0.0171 (8)	0.0201 (9)	−0.0022 (7)	−0.0002 (7)	−0.0029 (7)
C5	0.0307 (12)	0.0221 (10)	0.0214 (9)	−0.0049 (9)	0.0002 (9)	−0.0072 (8)
C6	0.0333 (13)	0.0273 (11)	0.0167 (9)	−0.0052 (10)	−0.0022 (8)	−0.0059 (8)
C7	0.0228 (10)	0.0236 (10)	0.0144 (8)	−0.0009 (8)	−0.0010 (7)	−0.0028 (7)
C8	0.0281 (11)	0.0293 (12)	0.0131 (7)	0.0006 (8)	−0.0008 (8)	0.0000 (8)
C9	0.0319 (12)	0.0269 (11)	0.0161 (8)	0.0014 (9)	−0.0001 (8)	0.0049 (8)
C10	0.0253 (10)	0.0197 (9)	0.0168 (8)	0.0007 (8)	0.0014 (7)	0.0029 (7)
C11	0.0165 (8)	0.0179 (8)	0.0134 (7)	−0.0003 (6)	0.0013 (6)	−0.0006 (6)
C12	0.0147 (7)	0.0156 (8)	0.0149 (7)	−0.0006 (6)	0.0012 (6)	−0.0008 (6)
O20	0.0445 (16)	0.0375 (14)	0.081 (2)	−0.0003 (12)	0.0101 (16)	−0.0038 (15)
C20	0.0370 (15)	0.0287 (13)	0.0288 (12)	0.0012 (11)	0.0023 (11)	0.0038 (10)
O30	0.317 (15)	0.317 (15)	0.040 (5)	0.000	0.000	0.000

### Geometric parameters (Å, °)

**Table:**

Nd1—F1^i^	2.3348 (15)	C1—C2	1.407 (3)
Nd1—F1	2.3348 (15)	C1—H1	0.9500
Nd1—O4	2.5328 (19)	C2—C3	1.373 (4)
Nd1—O4^i^	2.5328 (19)	C2—H2	0.9500
Nd1—O1	2.5574 (18)	C3—C4	1.406 (3)
Nd1—O1^i^	2.5574 (18)	C3—H3	0.9500
Nd1—O5	2.5648 (19)	C4—C12	1.398 (3)
Nd1—O5^i^	2.5648 (19)	C4—C5	1.437 (3)
Nd1—O2	2.5650 (18)	C5—C6	1.355 (4)
Nd1—O2^i^	2.5650 (18)	C5—H5	0.9500
Cr1—F1^ii^	1.8815 (15)	C6—C7	1.431 (4)
Cr1—F1	1.8815 (15)	C6—H6	0.9500
Cr1—N2	2.0551 (17)	C7—C11	1.401 (3)
Cr1—N2^ii^	2.0551 (17)	C7—C8	1.410 (4)
Cr1—N1	2.0616 (19)	C8—C9	1.371 (4)
Cr1—N1^ii^	2.0616 (19)	C8—H8	0.9500
N1—C1	1.326 (3)	C9—C10	1.403 (3)
N1—C12	1.367 (3)	C9—H9	0.9500
N2—C10	1.332 (3)	C10—H10	0.9500
N2—C11	1.367 (3)	C11—C12	1.425 (3)
N3—O3	1.225 (3)	O20—C20	1.416 (4)
N3—O2	1.268 (3)	O20—H20	0.8400
N3—O1	1.273 (3)	C20—H20A	0.9800
N4—O6	1.226 (3)	C20—H20B	0.9800
N4—O4	1.267 (3)	C20—H20C	0.9800
N4—O5	1.271 (3)		
			
F1^i^—Nd1—F1	72.09 (7)	Cr1—F1—Nd1	168.74 (8)
F1^i^—Nd1—O4	140.40 (6)	C1—N1—C12	118.15 (19)
F1—Nd1—O4	123.46 (5)	C1—N1—Cr1	129.31 (15)
F1^i^—Nd1—O4^i^	123.46 (5)	C12—N1—Cr1	112.52 (14)
F1—Nd1—O4^i^	140.40 (6)	C10—N2—C11	118.83 (19)
O4—Nd1—O4^i^	70.35 (9)	C10—N2—Cr1	128.39 (16)
F1^i^—Nd1—O1	82.88 (5)	C11—N2—Cr1	112.61 (13)
F1—Nd1—O1	75.88 (6)	O3—N3—O2	121.8 (2)
O4—Nd1—O1	134.02 (6)	O3—N3—O1	121.4 (2)
O4^i^—Nd1—O1	71.16 (7)	O2—N3—O1	116.77 (19)
F1^i^—Nd1—O1^i^	75.88 (6)	O6—N4—O4	121.5 (2)
F1—Nd1—O1^i^	82.87 (5)	O6—N4—O5	122.1 (2)
O4—Nd1—O1^i^	71.16 (7)	O4—N4—O5	116.4 (2)
O4^i^—Nd1—O1^i^	134.02 (6)	N3—O1—Nd1	96.72 (13)
O1—Nd1—O1^i^	153.69 (9)	N3—O2—Nd1	96.50 (13)
F1^i^—Nd1—O5	132.47 (6)	N4—O4—Nd1	97.01 (14)
F1—Nd1—O5	73.85 (5)	N4—O5—Nd1	95.39 (13)
O4—Nd1—O5	50.07 (6)	N1—C1—C2	122.3 (2)
O4^i^—Nd1—O5	103.91 (6)	N1—C1—H1	118.9
O1—Nd1—O5	119.27 (6)	C2—C1—H1	118.9
O1^i^—Nd1—O5	67.84 (6)	C3—C2—C1	119.6 (2)
F1^i^—Nd1—O5^i^	73.85 (5)	C3—C2—H2	120.2
F1—Nd1—O5^i^	132.47 (6)	C1—C2—H2	120.2
O4—Nd1—O5^i^	103.91 (6)	C2—C3—C4	119.4 (2)
O4^i^—Nd1—O5^i^	50.07 (6)	C2—C3—H3	120.3
O1—Nd1—O5^i^	67.84 (6)	C4—C3—H3	120.3
O1^i^—Nd1—O5^i^	119.27 (6)	C12—C4—C3	117.3 (2)
O5—Nd1—O5^i^	151.57 (9)	C12—C4—C5	118.5 (2)
F1^i^—Nd1—O2	125.40 (5)	C3—C4—C5	124.2 (2)
F1—Nd1—O2	71.02 (5)	C6—C5—C4	121.3 (2)
O4—Nd1—O2	93.81 (6)	C6—C5—H5	119.4
O4^i^—Nd1—O2	71.14 (6)	C4—C5—H5	119.4
O1—Nd1—O2	49.98 (6)	C5—C6—C7	120.9 (2)
O1^i^—Nd1—O2	135.60 (6)	C5—C6—H6	119.6
O5—Nd1—O2	70.66 (6)	C7—C6—H6	119.6
O5^i^—Nd1—O2	104.72 (6)	C11—C7—C8	117.2 (2)
F1^i^—Nd1—O2^i^	71.02 (5)	C11—C7—C6	118.6 (2)
F1—Nd1—O2^i^	125.40 (5)	C8—C7—C6	124.2 (2)
O4—Nd1—O2^i^	71.14 (6)	C9—C8—C7	119.5 (2)
O4^i^—Nd1—O2^i^	93.81 (6)	C9—C8—H8	120.2
O1—Nd1—O2^i^	135.60 (6)	C7—C8—H8	120.2
O1^i^—Nd1—O2^i^	49.98 (6)	C8—C9—C10	120.0 (2)
O5—Nd1—O2^i^	104.72 (6)	C8—C9—H9	120.0
O5^i^—Nd1—O2^i^	70.66 (6)	C10—C9—H9	120.0
O2—Nd1—O2^i^	161.92 (8)	N2—C10—C9	121.6 (2)
F1^ii^—Cr1—F1	91.41 (8)	N2—C10—H10	119.2
F1^ii^—Cr1—N2	97.93 (7)	C9—C10—H10	119.2
F1—Cr1—N2	90.16 (7)	N2—C11—C7	122.8 (2)
F1^ii^—Cr1—N2^ii^	90.16 (7)	N2—C11—C12	116.64 (18)
F1—Cr1—N2^ii^	97.93 (7)	C7—C11—C12	120.5 (2)
N2—Cr1—N2^ii^	168.43 (10)	N1—C12—C4	123.2 (2)
F1^ii^—Cr1—N1	89.75 (7)	N1—C12—C11	116.78 (19)
F1—Cr1—N1	170.49 (6)	C4—C12—C11	120.0 (2)
N2—Cr1—N1	80.33 (7)	C20—O20—H20	109.5
N2^ii^—Cr1—N1	91.51 (7)	O20—C20—H20A	109.5
F1^ii^—Cr1—N1^ii^	170.49 (6)	O20—C20—H20B	109.5
F1—Cr1—N1^ii^	89.75 (7)	H20A—C20—H20B	109.5
N2—Cr1—N1^ii^	91.50 (7)	O20—C20—H20C	109.5
N2^ii^—Cr1—N1^ii^	80.33 (7)	H20A—C20—H20C	109.5
N1—Cr1—N1^ii^	90.66 (10)	H20B—C20—H20C	109.5

Symmetry codes: (i) −*y*+1, −*x*+1, −*z*+1/2; (ii) *y*−1/2, *x*+1/2, −*z*+1/2.

### Hydrogen-bond geometry (Å, °)

**Table:**

*D*—H···*A*	*D*—H	H···*A*	*D*···*A*	*D*—H···*A*
O20—H20···O20^iii^	0.84	1.89	2.700 (4)	161.

Symmetry codes: (iii) *y*, −*x*+1/2, *z*.
